# Effect of natural products on the production and activity of *Clostridium difficile* toxins *in vitro*

**DOI:** 10.1038/s41598-018-33954-2

**Published:** 2018-10-24

**Authors:** Niloufar Roshan, Thomas V. Riley, Daniel R. Knight, Katherine A. Hammer

**Affiliations:** 1 School of Biomedical Sciences (M504), The University of Western Australia, 35 Stirling Hwy, Crawley, Western Australia 6009 Australia; 2 Division of Microbiology, PathWest Laboratory Medicine, Queen Elizabeth II Medical Centre, Nedlands, Western Australia 6009 Australia; 3 School of Veterinary & Life Sciences, Murdoch University, Murdoch, Western Australia 6150 Australia; 4 School of Medical & Health Sciences, Edith Cowan University, Joondalup, Western Australia 6027 Australia

## Abstract

*Clostridium difficile* infection is a toxin-mediated disease of the colon. *C*. *difficile* virulence is primarily attributed to the production of toxin A and toxin B; thus this study was aimed to investigate the effect of a range of natural products on the production and activity of *C*. *difficile* toxins *in vitro*. Twenty-two natural products were investigated against four *C*. *difficile* strains. The activity of products against toxins was determined using Vero and HT-29 cells cytotoxicity and neutral red uptake assays. The indirect effect of products on toxin-mediated cytotoxicity was determined using the same cell lines. The effect of seven products on toxin production by *C*. *difficile* was determined using ELISA. Zingerone (0.3 mg/ml) protected both cell lines from *C*. *difficile* cytopathic effects, confirmed by the neutral red uptake assay (*P* < 0.05). Three *Leptospermum* honeys (4% w/v), fresh onion bulb extract (12.5% v/v) and *trans*-cinnamaldehyde (0.005% v/v) all reduced toxin production and activity significantly (*P* ≤ 0.023). Garlic clove powder (4.7 mg/ml) only reduced toxin activity (*P* ≤ 0.047). Overall, several natural products had activity against *C*. *difficile* toxins *in vitro* encouraging further investigation against *C*. *difficile* toxins *in vivo*.

## Introduction

*Clostridium difficile* infection (CDI) is one of the most important healthcare-associated infections worldwide and a major cause of morbidity and mortality in both the hospital and community settings^1^. Typically, CDI occurs following the disruption of normal enteric flora, usually post-antimicrobial exposure, leading to the proliferation and germination of *C*. *difficile* spores into vegetative cells, resulting in toxin production in the intestine^2^. Toxin A (TcdA, 308 kiloDaltons, kDa) and toxin B (TcdB, 270 kDa) belong to the large clostridial glucosylating family of toxins and are the major virulence factors of this species^3^. Despite similar enzymatic activities, TcdA and TcdB have different cellular receptors, *in vivo* potency and immunological response, and recent studies of isogenic toxin mutants in hamster and piglet models provided convincing evidence that toxin B alone is essential for CDI^3,4^. Secretion of these potent toxins within the gastrointestinal tract causes actin disassembly, enterocyte apoptosis, the breakdown of epithelial tight junctions and an overall loss of epithelial integrity^3^. This pathophysiological cascade results in extensive colonic inflammation, epithelial tissue damage and a rapid loss of fluid into the intestinal lumen, manifesting as characteristic watery diarrhoea but may also develop into fatal sequelae including pseudomembranous and fulminant colitis^3,4^. *In vitro* cellular apoptosis is seen clearly in the characteristic “cell rounding” phenotype or cytopathic effect (CPE). Some *C*. *difficile* lineages, most notably the hypervirulent NAP1/027 epidemic strain, also produce a third toxin known as binary toxin (CDT), however, there is still little known about the role of this toxin in virulence^5^.

For the last three decades, CDI has been managed with the conventional antimicrobials metronidazole (for mild to moderate CDI) and vancomycin (for severe CDI)^6^. Despite retaining good *in vitro* efficacy against *C*. *difficile*, several issues remain surrounding the use of these agents. Increasingly, they are associated with unacceptably high rates of CDI recurrence, reduced efficacy *in vitro* and minimal inhibitory concentration (MIC) creep, particularly with metronidazole, and with vancomycin, there is a risk of selection for acquired glycopeptide resistance in Gram-positive pathogens^7–9^. Fidaxomicin became available for treatment of CDI in 2011. It has a narrower spectrum of activity compared to vancomycin and metronidazole, and thus may have has less impact on gut microbiota. However, its high cost compared to either metronidazole or vancomycin has restricted its use^10^.

Medicinal plants remain the primary source of treatment for several diseases in rural areas of many developing countries^11^. Plant-derived compounds are considered by some consumers as a safer, less toxic and more environmentally-friendly option compared to conventional therapies. They are typically multi-component in nature and the components contain different functional groups. As such, their antimicrobial activity often relates to multiple mechanisms; hence, unlike conventional antimicrobials, organisms are less likely to develop resistance^12^. We have shown recently that several natural products have antimicrobial activity against *C*. *difficile in vitro*^13^. Given that CDI is a toxin-mediated disease, the purpose of the present study was to investigate the effect of those previously tested natural products, and corresponding pure substances, on toxin production and activity in *C*. *difficile in vitro*. The anti-toxin activity of natural products and their impact on toxin-mediated cytotoxicity were assessed using tissue culture and cytotoxicity assays. ELISA was performed to determine the effect of these products on the production of *C*. *difficile* toxins.

## Results

### Antimicrobial susceptibility assay

The MICs of natural products (Table 1)^14^ and antimicrobial controls for the four strains of *C*. *difficile* have previously been published^13^ and are shown in Table S1. The only exception was the two extra *Leptospermum* honeys A and B as listed in Table 1. Both honeys A and B showed an MIC of 16% v/v, which was two-fold lower than previously tested *Leptospermum* honey C.

**Table 1:** Range of natural products used in this study.

Product	Concentration	Ingredient information	Supplier
**Raw products**			
Fresh garlic bulb	1 ml/ml	None	—
Fresh onion bulb	1 ml/ml	None	—
Fresh ginger rhizome	1 ml/ml	None	—
*Leptospermum* honey (MGO 514+) (A)	100% pure Manuka honey	None	Barnes Naturals, Richlands, QLD, Australia
*Leptospermum* honey (MGO 263+) (B)	100% pure Manuka honey	None	Barnes Naturals
*Leptospermum* honey (MGO 263+) (C)	100% pure Manuka honey	None	Barnes Naturals
Garlic clove powder	1 mg/mg	None	Spencers, Fremantle, WA, Australia
Ginger rhizome powder	1 mg/mg	None	Spencers
Cinnamon root powder	1 mg/mg	None	Spencers
Turmeric root powder	1 mg/mg^_^	None	Spencers
**Processed products**			
Garlic tablet	Garlic (*Allium sativum*) extract equivalent to dry bulb 10,000 mg	No added yeast, starch, gluten, lactose, sugar, artificial colours or flavours, artificial sweeteners or preservatives, dairy products or animal-derived products.	Nature’s Own, Virginia, QLD, Australia
Ginger tablet	1000 mg	No added yeast, starch, gluten, lactose, sugar, artificial colours or flavours, artificial sweeteners or preservatives, dairy products or animal-derived products.	Nature’s Own
Cinnamon tablet	Cinnamon (*Cinnamomum cassia*) stem bark inner powder 1000 mg	No added lactose, egg, gluten, soy, yeast, artificial colours and artificial flavours.	Swisse, Collingwood, VIC, Australia
Turmeric tablet	1000 mg	Turmeric powder 77%, cellulose microcrystalline, povidone, silica colloidal anhydrous, magnesium stearate, croscarmelloose sodium.	Nature’s Way, Warriewood, NSW, Australia
**Processed products**			
Artichoke capsule	Herbal extract equivalent *to Cynara scolymus* fresh leaf 60000 mg	No added lactose, starch, added sugars and salt, artificial colourings and flavourings or preservatives.	Nature’s Sunshine, Baulkham hills, NSW, Australia
Coconut oil capsule	100% coconut oil	Capsule shell (gelatin, glycerol, water)	Nature’s Way
Peppermint oil	100% pure, *Mentha X piperita* leaf 1 ml/ml	None	Oil Garden, Springvale, VIC, Australia
Aloe vera gel	99% pure *Aloe Barbadensis*	Allantoin (found in Comfrey root), carbomer, disodium edetate, sodium hydroxyl methylglycinate, grapefruit seed extract	Plunkett’s, Warriewood, NSW, Australia
**Pure compounds**			
Allicin	180 mg allicin extract (garlic bulb)	Vegetarian capsule (hypromellose, water), maltodextrin, gum acacia, Made without corn, wheat, gluten, yeast, soy, sugar, dairy, artificial colors or flavors	Vcaps, Chicago, IL, USA.
*trans*-Cinnamaldehyde	Purity >98.5%	None	Sigma Aldrich, Saint Louis, MO, USA
Menthol	Purity >98.5%	None	Sigma Aldrich
Zingerone	Purity >96%	None	Sigma Aldrich

MGO, methylglyoxal.

(Table extracted from Roshan *et al*. 2018).

### Toxin protection assay

**Figure 1:**
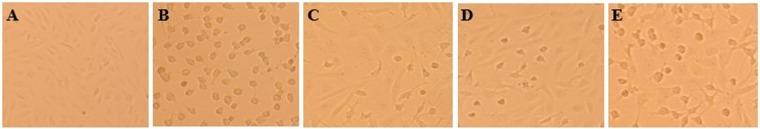
Protection from cytopathic effect on Vero cells using microscopy (*C*. *difficile* NCTC 13366 culture filtrate and zingerone were incubated at 37 °C for 2 h prior to being added to Vero cell monolayers). (**A**) No culture filtrate; (**B**) culture filtrate only; (**C**) zingerone (1.2 mg/ml); (**D**) zingerone (0.6 mg/ml); (**E**) zingerone (0.3 mg/ml); Light microscopy ×40, Scale: 50 µm.

**Figure 2:**
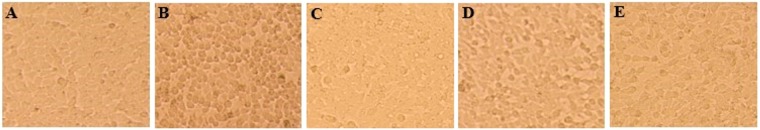
Protection from cytopathic effect on HT-29 cells using microscopy (*C*. *difficile* NCTC 13366 culture filtrate and zingerone were incubated at 37 °C for 2 h prior to being added to HT-29 cell monolayers). (**A**) No culture filtrate; (**B**) culture filtrate only; (**C**) zingerone (1.2 mg/ml); (**D**) zingerone (0.6 mg/ml); (**E**) zingerone (0.3 mg/ml); Light microscopy ×40, Scale: 50 µm.

**Figure 3:**
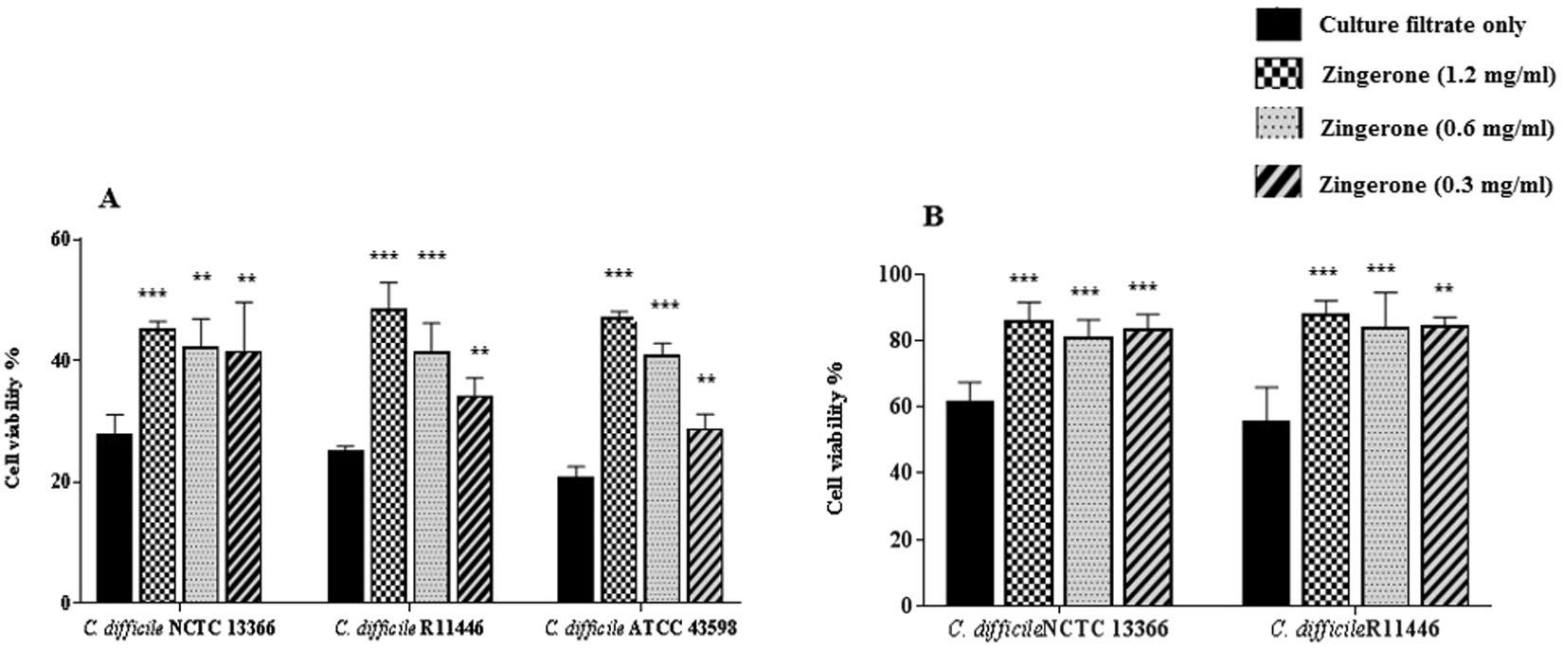
Cell viability determined by neutral red uptake assay. (Culture filtrates and treatments were incubated 2 h prior to being added to the cells); (**A**) Vero cells; (**B**) HT-29 cells; Statistical significance: *P < 0.05, **P < 0.01, ***P < 0.001 compared to culture filtrate control.

The supernatants from the three toxigenic strains demonstrated CPE in both Vero and HT-29 cells. However, only minor CPE was observed with *C*. *difficile* ATCC 43598 and HT-29 cells. This was shown as cell rounding, membrane blebbing and loss of adhesion. The toxin titres for each of the three toxigenic *C*. *difficile* strains causing approximately 90% CPE were determined over three independent experiments. A modal toxin titre was selected for each strain (Table 2). The highest concentrations of each antimicrobial agent showing no effect on cells are shown in Table S2. In addition, the pH of the growth medium with and without the addition of each compound was measured to determine whether there was a deviation in pH that could interfere in the assay. No significant deviation was observed. When culture filtrates were incubated for 2 h with antimicrobial agents, only zingerone protected Vero cells against any cytotoxic effect visually. This was confirmed by a neutral red uptake assay (*P* ≤ 0.007) (Figs 1, S1 and S2). Also, when Vero cells were pre-incubated with zingerone for 2 h prior to the addition of culture filtrates, a similar protective effect was observed. However, when zingerone and culture filtrates were transferred to the wells containing Vero cell monolayers together, and with no prior incubation, no protection was observed. Zingerone showed a similar protective effect against toxin activity on HT-29 cell monolayers (Figs 2, 3 and S3).

**Table 2:** *C*. *difficile* toxin titre required to achieve 90% CPE on Vero and HT-29 cells after 24 h incubation at 37 °C with 5% CO_2_.

*C*. *difficile* strain	Modal toxin titre (range)	
	Vero cells	HT-29 cells
NCTC 13366	1:524,288 (1:524,288 – 1:2,097152)	1:262,144 (131,072-524,288)
R11446	1:8,192 (1:2,048 – 1:8,192)	1:8,192 (4,096-8,16,384)
ATCC 43598	1:512 (1:512 – 1:1,024)	1:8 (1:8-1:32)

### Indirect effect of natural products on toxin-mediated cytotoxicity

**Figure 4:**
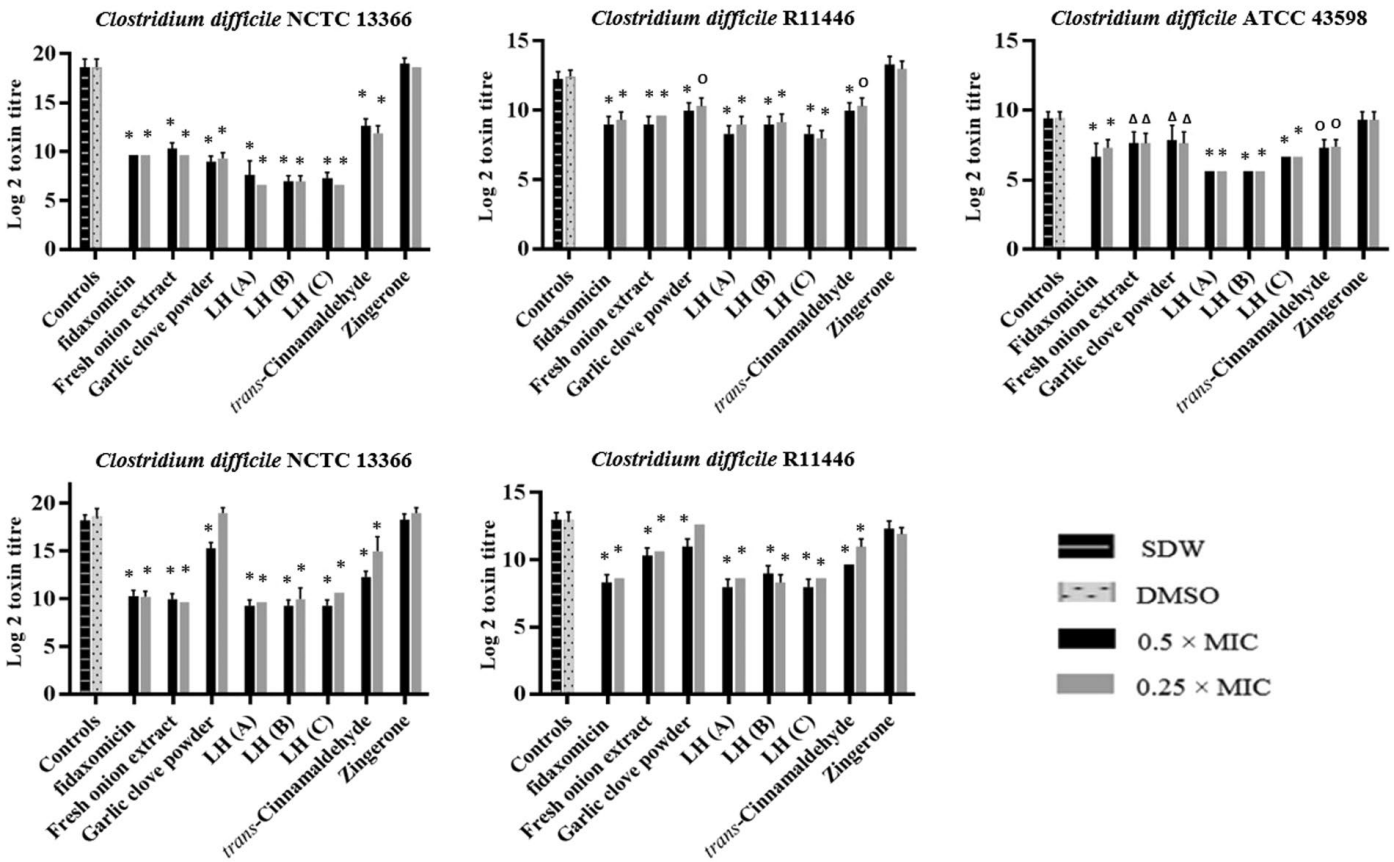
Indirect effect of treatments on *C*. *difficile* cytotoxicity on (**A**) Vero cells and (**B**) HT-29 cells. Concentrations of agents used in the assay: Fresh onion bulb extract (25% and 12.5% v/v); Garlic clove powder (4.7 and 2.3 mg/ml); *Leptospermum* honey (**A**), (**B**) and (**C**) (8% and 4% v/v); *trans*-Cinnamaldehyde (0.01% and 0.005 v/v); Zingerone (4.7 and 2.3 mg/ml); Fidaxomicin (0.06 µg/ml). Statistical significance: ^∆^*P* < 0.05, ^Ο^*P* < 0.01, *P < 0.001 compared to controls; *LH, *Leptospermum* honey; SDW, sterile distilled water; DMSO, dimethyl sulfoxide.

The effect of all products on toxin-mediated cytotoxicity on Vero cells was examined for *C*. *difficile* NCTC 13366 and R44116 (Table S3), and products showing a reduction in CPE were further tested against *C*. *difficile* ATCC 43598. Exposure to six of the 22 natural products significantly reduced cytotoxicity with Vero cells compared to untreated culture filtrate of the three toxigenic strains of *C*. *difficile* (*P* ≤ 0.047) (Fig. 4). The cytotoxicity titres for *C*. *difficile* cultures incubated for 48 h with 0.5 × and 0.25 × MICs of fresh onion bulb extract, garlic clove powder, *trans*-cinnamaldehyde and the three *Leptospermum* honeys were reduced by ≥70% compared to the untreated controls. Similar findings were observed for HT-29 cells (*P* ≤ 0.001), with the exception of garlic powder at 0.25 × MIC which did not show any reduction in CPE.

### Effect of treatments on C. difficile toxin production

**Figure 5:**
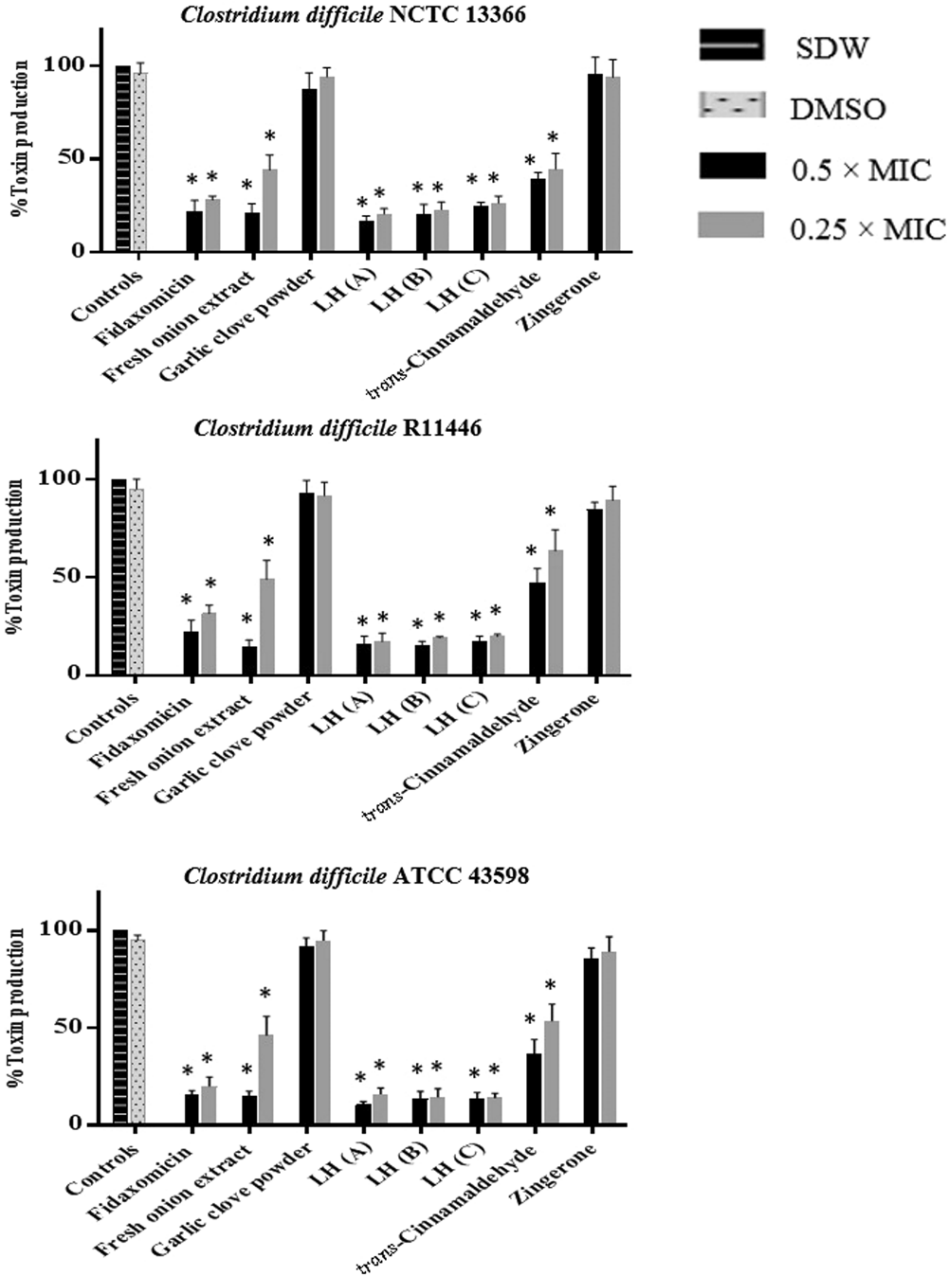
Effect of treatments on *C*. *difficile* toxin production. Concentrations of agents used in the assay: Fresh onion bulb extract (25% and 12.5% v/v); Garlic clove powder (4.7 and 2.3 mg/ml); *Leptospermum* honey (**A**,**B**) and (**C**) (8% and 4% v/v); *trans*-Cinnamaldehyde (0.01% and 0.005 v/v); Zingerone (4.7 and 2.3 mg/ml); Fidaxomicin (0.06 µg/ml). Statistical significance: ^∆^*P* < 0.05, ^Ο^*P* < 0.01, *P < 0.001 compared to controls; *LH, *Leptospermum* honey; SDW, sterile distilled water; DMSO, dimethyl sulfoxide.

Seven of the 22 products that showed activity in either direct or indirect toxin activity assays were selected to determine their effect on toxin production by the three toxigenic *C*. *difficile* strains using ELISA. Toxin production for *C*. *difficile* strains in the presence of treatments relative to untreated cultures is shown in Fig. 5. Fresh onion bulb extract, *trans*-cinnamaldehyde and the three *Leptospermum* honeys significantly reduced toxin production by all three toxigenic strains compared to the untreated controls (*P* < 0.001). At 48 h, these products inhibited toxin production by ≥40% in the toxigenic strains.

## Discussion

Toxins are the main virulence factors of *C*. *difficile*, which initiates gastrointestinal disease by causing inflammation and mucosal damage in the intestine^15^. Therefore, investigating toxin-active therapeutic agents with low risk of developing antimicrobial resistance is of great interest in either treating or preventing CDI^8^. In this study, the effect of a range of natural products, and the corresponding pure substance, on toxin activity of *C*. *difficile* was examined using Vero and HT-29 cells. Similar results were observed with both cell lines. The toxin protection study showed that the ginger component zingerone protected cells against the effect of *C*. *difficile* toxin. In this assay, protection against toxin activity was observed when culture filtrates and zingerone were incubated together for 2 h prior to being added to the cells, as well as when the cells were pre-incubated with zingerone prior to addition of culture filtrates. However, no significant protection was observed when toxin filtrates and zingerone were added simultaneously to wells containing either Vero or HT-29 cells. Hence, it is likely that zingerone provides protection against the action of *C*. *difficile* toxin by blocking either the toxin-binding sites on toxin molecules or the host cell receptors. In an earlier study by Chen *et al*., the effect of ginger and its bioactive components on inhibition of enterotoxigenic *Escherichia coli* heat-labile enterotoxin (LT)-induced diarrhea in mice was investigated. Ginger extract inhibited fluid accumulation in the ileal loops of mice by blocking the binding of LT to G_M1_ cell-surface receptor. Further investigation of biologically-active components showed that zingerone was the active constituent responsible for the anti-diarrhoeal effect of ginger^16^.

With the indirect toxin-mediated cytotoxicity assay and ELISAs, several products such as the three *Leptospermum* honeys, fresh onion bulb extract and *trans*-cinnamaldehyde at 0.5 and 0.25 × MIC, showed a reduction in both toxin production and activity. Garlic clove powder at 0.5 and 0.25 × MIC showed a reduction in toxin activity with Vero cells and at 0.5 × MIC with HT-29 cells. Also, the ELISAs did not indicate any significant decrease in toxin production with garlic powder compared to the untreated controls. Previously, *trans*-cinnamaldehyde (0.38 mM; 0.05 mg/mL) reduced both *C*. *difficile* toxin production and activity *in vitro*^17^. In a study by Upadhyay *et al*., *trans*-cinnamaldehyde (0.5 mM) down-regulated *hly* and *prfA* genes coding for toxin production and a transcriptional regulator in *Listeria monocytogenes*^18^. The activity of several antimicrobials such as fidaxomicin, its major metabolite (OP-1118), metronidazole and vancomycin against *C*. *difficile* toxin has been investigated previously^19^. That study showed that both fidaxomicin and OP-1118 at 0.25 × MIC inhibited toxin production by ≥60% following 1 week of culture. Also, a near complete inhibition of toxin gene expression was observed with both fidaxomicin and OP-1118 at 2 and 2.5 × MIC, respectively. No inhibitory effect on *C*. *difficile* toxin production or gene expression was reported for either metronidazole or vancomycin^19^. In our study, fidaxomicin was used as an antimicrobial control and showed a reduction in both *C*. *difficile* toxin production and activity.

Notwithstanding the protective effect of zingerone against *C*. *difficile* toxin observed in the toxin protection assay, zingerone did not show any effect in either the indirect toxin-mediated cytotoxicity assay or toxin production assays. Theoretically, zingerone has a relatively rapid bactericidal action and its activity may degrade over time, although there are no published literature to support this hypothesis. In indirect toxin-mediated cytotoxicity and toxin production assays, treatments were not exposed directly to the toxin and it would have had taken some time for *C*. *difficile* to initiate toxin production. By the time the cells had started to produce toxin, the activity of zingerone may have been minimal. Moreover, since gene expression for toxin production occurs during the late log-phase, the addition of the compound during this stage could determine the effect of zingerone on toxin gene expression. This will be studied in a series of experiments in the future.

Food-grade and plant-derived compounds are used commonly among people who practice self-health care^10^. Many consumers turn to natural products assuming that natural is synonymous with safe. This concept is not necessarily true as side effects are expected with some^20^. Some natural products such as *trans*-cinnamaldehyde are toxic to humans if consumed in large quantities^21^. However, many natural products, including those used in this study, have “generally recognised as safe” (GRAS) status with the US Food and Drug Administration. This means that when consumed in moderate amounts they are unlikely to pose a health risk^21^. In addition, further studies on the chemical composition of these products, as distinct from pure substances, will be of importance. Until such studies are done, the results from investigations of “natural products’ should be interpreted with caution.

## Conclusions

Overall, this study highlights the activity of a number of food-grade and plant-derived products against *C*. *difficile* toxins *in vitro*. These results suggest that several natural products may have the potential to be considered as either alternative or complementary treatment options for CDI. Applying anti-virulence strategies such as disruption of toxin production and toxin-mediated pathology in patients with CDI has the potential to be an effective approach in either controlling or treating infection. However, further studies are required to investigate the mechanistic basis for the anti-toxin activity observed as well as to determine their potential benefits *in vivo*.

## Material and Methods

### Bacterial strains

The following four *Clostridium difficile* strains were used in this study: non-toxigenic *C*. *difficile* ATCC 700057 (A^−^B^−^CDT^−^), *C*. *difficile* NCTC 13366 (PCR ribotype (RT) 027, A^+^B^+^CDT^+^), *C*. *difficile* R11446 (RT 014, A^+^B^+^CDT^−^, clinical isolate) and *C*. *difficile* ATCC 43598 (RT 017, A^−^B^+^CDT^−^). Strains were obtained from the School of Biomedical Sciences at The University of Western Australia and PathWest Laboratory Medicine, WA.

### Antimicrobial agents

Twenty-two natural products were selected for investigation based on historical evidence, their current popularity, and feasibility (Table 1). The products were categorized into three broad groups; raw products usually consumed as food, processed products taken as health supplements and the active pure compounds found in these products. The raw products (n = 10) were purchased from a supermarket in Perth, Western Australia (WA) (Table 1) while the *Leptospermum* honeys were kindly provided by Capilano Company, Bayswater, WA. Processed and pure products (n = 12) were purchased from pharmacies and health food stores in Perth, WA, or from the manufacturers (Table 1). The products were prepared for testing as previously described^13^. Fidaxomicin and vancomycin were used as antimicrobial controls in susceptibility testing and fidaxomicin was used as an effective-antimicrobial control in tissue culture and ELISA assays. Stock solutions of products in the form of powder or oil were prepared in 20% dimethyl sulfoxide (DMSO). Further dilutions were performed in sterile distilled water (SDW) for all products. Prior to testing, raw products were filtered through a 0.22 µm-pore size Millipore filter to remove any microbial contamination, however, the processed products were not filtered as they were assumed to have had gone through a sterilization process during manufacturing.

### Determination of minimum inhibitory concentration (MIC)

Minimum inhibitory concentrations (MIC) for natural products and comparator antimicrobial agents were determined by the broth microdilution method as previously described^13^. Doubling dilutions of 20% DMSO were prepared and tested in parallel (solvent control), and fidaxomicin and vancomycin were included as antibiotic controls. The previously determined MICs for these products against the stationary phase *C*. *difficile* were used in this study.

### Toxin protection assay

#### Tissue culture

The toxin protection assay was performed as described previously^8^ with slight modification. The Vero (African green monkey kidney) and HT-29 (human colon carcinoma) cells were passaged in a 96-multiwell plate and incubated for 24 h prior to use. To prepare confluent monolayers, Vero and HT-29 cells were dispensed into 96-well trays at 6 × 10^4^ cells/well and 5 × 10^5^ cells/well, respectively. Vero cells were passaged and maintained in Dulbecco’s Modified Eagles Medium (DMEM). DMEM supplemented with 10% (DMEM-GM) and 2% foetal bovine serum (DMEM-MM) were used as growth and maintenance medium, respectively. Both DMEM-GM and DMEM-MM also contained 200 µg/ml vancomycin and 100 µg/ml streptomycin sulfate. HT-29 was passaged and maintained as above, except that the medium also contained glucose (6 g/l).

#### Culture filtrate preparation

*C*. *difficile* strains were cultured on blood agar for 48 h, from which suspensions equivalent in turbidity to a 2 McFarland standard were prepared in 0.85% saline. A volume of 100 µl of each suspension was inoculated into a 10 ml pre-reduced brain heart infusion broth (BHIB). Following 5 h of anaerobic incubation, the broth cultures were aseptically sealed and incubated for a further 5 days at 37 °C in a Thermocube incubator (Bioline) with shaking at 120 revolutions per minute (rpm). After 5 days of incubation, cultures were centrifuged at 4,000 × *g* for 5 min and the supernatant containing toxin was passed through a 0.22 µm-pore size Millipore filter to remove cells and debris.

#### Toxin titration

Serial two-fold dilutions of culture filtrate in DMEM-MM were prepared with a total volume of 100 µl per well and were added to the cells. The 90% cytotoxicity titre was determined visually using an inverted microscope (Olympus Corporation, Tokyo, Japan) at ×400 magnification. All titrations were performed three times.

#### Preparation of natural products

Natural products were prepared as described above. Serial two-fold dilutions of natural products, fidaxomicin and DMSO were prepared in DMEM-MM and 100 µl volumes of each were added to Vero cell monolayers. The highest concentrations showing no visible effect on cell monolayers after 24 h incubation was used in the subsequent assay.

#### Incubation of products and culture filtrates

The highest concentrations of all products with no visible effect on cell monolayers, in addition to 2-fold and 4-fold lower concentrations, were used in this assay. Those products that showed protection against toxin on Vero cells were also tested on HT-29 cells. Supernatants from the three toxigenic *C*. *difficile* strains were diluted to 4-fold higher than the minimum dilution required to achieve 90% cell rounding to account for the possible variation and dilution factor. The culture filtrates and products were both diluted in DMEM-MM. To evaluate whether natural products reduced the activity of free toxin, diluted antimicrobial agents and supernatants were mixed at a ratio of 1:1 and incubated at 37 °C for 2 h. Aliquots of 100 µl of 1:1 antimicrobial agents: culture filtrates were added to 96-well microtiter plates containing Vero cell monolayers and incubated for 24 h at 37 °C in a CO_2_ incubator (Thermo Fisher Scientific, Waltham, MA USA) containing 5% CO_2_. Protection from CPE was evaluated with an inverted microscope and was verified with a neutral red uptake assay (see below).

Culture filtrates from non-toxigenic *C*. *difficile* ATCC 700057 and the three other toxigenic strains without the addition of antimicrobial agents were used as controls, with the three toxigenic strains showing a CPE but not the non-toxigenic strain. The assay was repeated on three different occasions. If protection was achieved by any of the products, they were further investigated to assess their likely mode of action, such as blocking the binding sites or inactivating the toxin. To investigate whether that product blocked cell binding sites, the cell monolayers were pre-incubated for 2 h with treatments at 2-fold higher than the desired concentrations at 37 °C with 5% CO_2_, followed by the addition of an equivalent amount of *C*. *difficile* culture filtrate. Lastly, to evaluate whether protection occurred immediately or pre-incubation was required, diluted antimicrobial agents and culture filtrates were mixed at a ratio of 1:1 and 100 µl of the mixture was immediately transferred to the wells containing cell monolayers.

### Neutral red uptake assay

A neutral red uptake assay was performed to determine cell viability and to confirm the visual perception of protection from CPE^21^. Following the visual evaluation of results after 24 h of incubation, the contents from each well were removed and a 100 µl aliquot of 40 µg/ml neutral red solution was added. Plates were incubated for 3 h at 37 °C with 5% CO_2,_ then washed with 150 µl of phosphate buffered saline (PBS pH 7.3). To elute the stain, 150 µl of neutral red destain solution was added to each well and the plates were placed on a shaker at 120 rpm for 10 min. The optical density of each well was measured at 540 nm using an xMark^TM^ microplate spectrophotometer (Bio-Rad). Wells without cells served as blanks. The percentage of viable cells in wells containing treated and untreated culture filtrates was calculated relative to the untreated control cells, which were assumed to be 100% viable.

### Indirect toxin-mediated cytotoxicity assay

The indirect effect of all 22 products on the activity of *C*. *difficile* toxins was determined using the Vero cell cytotoxicity assay as described previously^22^. Those products showing any effect against toxin on Vero cells were further tested with HT-29 cells. Briefly, a bacterial suspension of 1 × 10^6^ cfu/ml was prepared for each strain in pre-reduced BHIB containing 0.5 × MIC and 0.25 × MIC of treatments. The final volume for each bacterial suspension was 5 ml after addition of treatments. After incubating the cultures for 48 h anaerobically at 35 °C, they were centrifuged at 4,000 *g* for 10 min and the supernatant filtered through a 0.22 µm pore-size membrane filter. The culture filtrates were diluted 1:100 prior to being added to the cell monolayer plates to reduce the gross effect of products on cells. The diluted culture filtrates were further serially diluted 1:2 in 96-well microtiter plates containing cell monolayers using DMEM-MM. The plates were incubated at 37 °C in 5% CO_2_ for 48 h and examined under an inverted microscope. The reciprocal of the highest dilution causing 90% cell rounding was expressed as the cytotoxicity titre. All the products were tested against *C*. *difficile* NCTC 13366 and *C*. *difficile* R11446 using Vero cells and those products showing a reduction in cytotoxicity titre were further tested against *C*. *difficile* ATCC 43598. Only *C*. *difficile* NCTC 13366 and *C*. *difficile* R11446 were used for the HT-29 cell line, as just a minor CPE was observed with *C*. *difficile* ATCC 43598 with those cells. Fidaxomicin was used as a positive control (reducing CPE) and culture filtrate from *C*. *difficile* ATCC 700057 was used as a negative control (showing no CPE) in this assay.

### Effect of products on toxin production

The amount of toxin in cultures grown in the presence of 0.5 × MIC and 0.25 × MIC of treatments showing activity in either toxin activity assays was determined relative to the amount of toxin in control cultures (grown without antimicrobials) using the *C*. *difficile* TOX A/B II^TM^ ELISA kit (TechLab®). Also, fidaxomicin was included as an antimicrobial control. The culture supernatant from *C*. *difficile* strains was prepared and filtered as described earlier. The culture filtrates were diluted and tested according to the manufacturer’s instructions. The spectrophotometer was blanked against air at a wavelength of 620 nm and the optical density (OD) was measured at 450 nm. Relative toxin production was expressed as a percentage of OD_450_ for treated culture filtrates over OD_450_ of untreated culture filtrates.

### Statistical analysis

All assays were repeated at least three times. A modal value was determined for MIC values obtained by the broth microdilution assay and for the cytotoxicity titres in the indirect cytotoxicity assay. The mean and standard deviation (SD) were calculated for the percentage of viable cells in the neutral red uptake assay and for toxin production in ELISA assays. Statistical differences between three or more sets of data were analysed using GraphPad Prism v.7 software using one-way analysis of variance (ANOVA) and nonparametric technique, followed by Tukey’s multiple comparison post-test if the *P* value was significant. Moreover, the Student’s 2-tailed *t*-test assuming unequal variance was used to determine whether there was a significant difference between two sets of data. Toxin titres in the indirect toxin study were converted to log_2_ values and titre reciprocals that were lower than 100 were converted to a lower titre reciprocal of 50 to enable analysis. *P* values of <0.05 were considered significant.

## Electronic supplementary material

S1, S2, S3
