# Supplementary material for: Effect of natural products on the production and activity of Clostridium difficile toxins in vitro
Source: Sci Rep. 2018 Oct 24;8:15735. doi: 10.1038/s41598-018-33954-2 (PMC6200812; doi:10.1038/s41598-018-33954-2)
Supplement: Supplementary file 1 — S1, S2, S3 [file 41598_2018_33954_MOESM1_ESM.pdf]

Tile: Effect of natural products on the production and activity of *Clostridium difficile* toxins *in vitro*

Authors:

Niloufar Roshan [a], Thomas V. Riley [a-d], Daniel R. Knight [c], Katherine A. Hammer [a]

Affiliations:

[a] School of Biomedical Sciences (M504), The University of Western Australia, 35 Stirling Hwy, Crawley, Western Australia, Australia, 6009.

[b] Division of Microbiology, PathWest Laboratory Medicine, Queen Elizabeth II Medical Centre, Nedlands, Western Australia, Australia, 6009.

[c] School of Veterinary & Life Sciences, Murdoch University, Murdoch, Western Australia, Australia, 6150.

[d] School of Medical & Health Sciences, Edith Cowan University, Joondalup, Western Australia, Australia, 6027.

***Correspondence:**

K. A. Hammer, School of Biomedical Sciences (M504), The University of Western Australia, 35 Stirling Hwy, Crawley, Western Australia, 6009.

Tel: +61 8 6457 2137; Fax: +61 8 9346 2912; Email: katherine.hammer@uwa.edu.au

Table S1. MICs of natural products against stationary-phase cultures of *C. difficile*.

**Antimicrobial agents**		**Primary solvent**	***C. difficile* NCTC 13366**	***C. difficile* R11446**	***C. difficile* ATCC 43598**	***C. difficile* ATCC 700057**
Raw products						
Fresh garlic bulb extract	MIC	-	0.8	0.8	0.8	0.8
% (v/v) [a]	MBC		50	50	50	50
Fresh ginger rhizome	MIC	-	>50	>50	>50	>50
extract % (v/v) [a]	MBC		>50	>50	>50	>50
Fresh onion bulb extract	MIC	-	>50	>50	>50	>50
% (v/v) [a]	MBC		>50	>50	>50	>50
Leptospermum honey (A)	MIC	SDW	16	16	16	16
% (w/v)[b**]	MBC		>32	>32	>32	>32
Leptospermum honey (B)	MIC	SDW	16	16	16	16
% (w/v)[b**]	MBC		>32	>32	>32	>32
Leptospermum honey (C)	MIC	SDW	32	32	32	32
% (w/v) [b**]	MBC		>32	>32	>32	>32
Garlic clove powder	MIC	20% DMSO	9.4	9.4	9.4	9.4
(mg/ml)[c]	MBC		>150	>150	>150	>150
Ginger rhizome powder	MIC	20% DMSO	>150	>150	>150	>150
(mg/ml)[c]	MBC		>150	>150	>150	>150
Cinnamon root powder	MIC	20% DMSO	75	75	75	75
(mg/ml)[c]	MBC		75	75	150	75
Turmeric root powder	MIC	20% DMSO	>150	>150	>150	>150
(mg/ml)[c]	MBC		>150	>150	>150	>150
Processed products						
Garlic tablet (mg/ml)[c]	MIC	20% DMSO	37.5	37.5	75	37.5
	MBC		>150	>150	>150	>150
Ginger tablet (mg/ml)[c]	MIC	20% DMSO	>150	>150	>150	>150
	MBC		>150	>150	>150	>150
Cinnamon tablet (mg/ml)[c]	MIC	20% DMSO	>150	>150	>150	>150
	MBC		>150	>150	>150	>150
Turmeric tablet (mg/ml)[c]	MIC	20% DMSO	>150	>150	>150	>150
	MBC		>150	>150	>150	>150
Artichoke capsule	MIC	20% DMSO	75	75	150	75
(mg/ml)[c]	MBC		>150	>150	>150	>150
Aloe vera gel % (w/v)[b]	MIC	20% DMSO	16	16	16	16
	MBC		>32	>32	>32	>32
Coconut oil % (v/v)[b]	MIC	20% DMSO	>32	>32	>32	>32
	MBC		>32	>32	>32	>32
Peppermint oil % (v/v)[b]	MIC	20% DMSO	8	8	8	8
	MBC		8	8	8	8
Pure compounds						
Allicin (mg/ml)[c]	MIC	20% DMSO	4.7	2.3	4.7	4.7
	MBC		>75	>75	>75	>75
trans-Cinnamaldehyde %	MIC	20% DMSO	0.02	0.02	0.02	0.02
(v/v)[b]	MBC		0.02	0.02	0.02	0.02
Menthol (mg/ml)[c]	MIC	20% DMSO	9.4	9.4	9.4	9.4
	MBC		18.8	18.8	18.8	18.8
Zingerone (mg/ml)[c]	MIC	20% DMSO	9.4	9.4	9.4	9.4
	MBC		9.4	18.8	9.4	9.4
Control						
Fidaxomicin (µg/ml)[d]	MIC	SDW	0.25	0.125	0.25	0.125
	MBC		0.25	0.125	0.25	0.125
Vancomycin (µg/ml)[d]	MIC	SDW	1	0.5	0.5	0.5
	MBC		>16	>16	>16	>16

MIC, minimum inhibitory concentration; MBC, minimum bactericidal concentration; SDW, sterile distilled water; MGO, methylglyoxal; ND, not done.

*Serial two-fold dilutions were performed in SDW for all the products.***Leptospermum* honey (A), MGO 263+; *Leptospermum* honey (B), MGO 263+; *Leptospermum* honey (C), MGO 263+.

Dilutions starting at a50, b32, c150, d16.

[1] In some instances concentrations could not be supra-MIC due to DMSO or not showing a MIC value in the range of concentration of treatments used. [2] For the products that showed an effect, the assay was repeated with two-fold and four-fold lower concentrations. Dash means no solvent. (Table extracted from Roshan et al., 2017).

Table S2. Highest concentration of antimicrobial agents with no effect on Vero and HT-29 cells.

**Antimicrobial agents**	**Concentrations**	
	**Vero cells**	**HT-29 cells**
Fresh garlic bulb extract % (v/v)	0.05	ND
Fresh ginger rhizome extract % (v/v)	0.8	ND
Fresh onion bulb extract % (v/v)	0.4	0.8
Leptospermum honey % (w/v) (A)	0.5	0.5
Leptospermum honey % (w/v) (B)	0.5	0.5
Leptospermum honey % (w/v) (C)	0.5	0.5
Garlic clove powder (mg/ml)	0.6	1.2
Ginger rhizome powder (mg/ml)	18.8	ND
Cinnamon root powder (mg/ml)	18.8	ND
Turmeric root powder (mg/ml)	18.8	ND
Garlic tablet (mg/ml)	2.3	ND
Ginger tablet (mg/ml)	18.8	ND
Cinnamon tablet (mg/ml)	18.8	ND
Turmeric tablet (mg/ml)	18.8	ND
Artichoke capsule (mg/ml)\	2.3	ND
Aloe vera gel % (v/v)	0.06	ND
Coconut oil % (v/v)	0.13	ND
Peppermint oil % (v/v)	0.03	ND
Allicin (mg/ml)	1.2	ND
trans-Cinnamaldehyde % (v/v)	0.01	0.004
Menthol (mg/ml)	0.4	ND
Zingerone (mg/ml)	1.2	1.2
Fidaxomicin (µg/ml)	1	0.5

ND, not done.

Table S3. Modal Cytotoxicity titre for *C. difficile* cells inoculated with 0.5 × MIC of antimicrobial agents.

**Antimicrobial agents**	**Vero cells**			**HT-29 cells**	
	C. difficile **NCTC 13366**	***C. difficile*** **R11446**	***C. difficile*** **ATCC 43598**	***C. difficile*** **NCTC 13366**	***C. difficile*** **R11446**
Fresh garlic bulb extract (0.4% v/v)	204800	1600	ND	ND	ND
Fresh ginger rhizome extract (50% v/v)	409600	3200	ND	ND	ND
Fresh onion bulb extract (25% v/v)	800***	200***	200*	800***	1600***
Leptospermum honey (8% w/v) (A)	100***	200***	< 100***	800***	400***
Leptospermum honey (8% w/v) (B)	100***	200***	< 100***	800***	400***
Leptospermum honey (8% w/v) (C)	100***	200***	100***	800***	200***
Garlic clove powder (4.7 mg/ml)	800***	400***	200*	51200***	1600***
Ginger rhizome powder (150 mg/ml)	204800	1600	ND	ND	ND
Cinnamon root powder (37.5 mg/ml)	204800	1600	ND	ND	ND
Turmeric root powder (150 mg/ml)	409600	3200	ND	ND	ND
Garlic tablet (18.8 mg/ml)	409600	3200	ND	ND	ND
Ginger tablet (150 mg/ml)	409600	3200	ND	ND	ND
Cinnamon tablet (150 mg/ml)	409600	1600	ND	ND	ND
Turmeric tablet (150 mg/ml)	409600	3200	ND	ND	ND
Artichoke capsule (37.5 mg/ml)\	409600	3200	ND	ND	ND
Aloe vera gel (8% v/v)	819200	3200	ND	ND	ND
Coconut oil (32% v/v)	409600	3200	ND	ND	ND
Peppermint oil (2% v/v)	409600	1600	ND	ND	ND
Allicin (1.2 mg/ml)	409600	3200	ND	ND	ND
trans-Cinnamaldehyde (0.01% v/v)	3200***	400**	200**	6400***	800***
Menthol (4.7 mg/ml)	409600	1600	ND	ND	ND
Zingerone (4.7 mg/ml)	409600	3200	800	51200***	6400***
Fidaxomicin (0.06 µg/ml)	800***	200***	100***	1600***	400***
SDW	409600	3200	800	409600	6400
DMSO (3.4% v/v)	409600	3200	800	409600	6400

ND, not done. Statistical significance: *<0.05, **<0.01, ***<0.001 compared to SDW

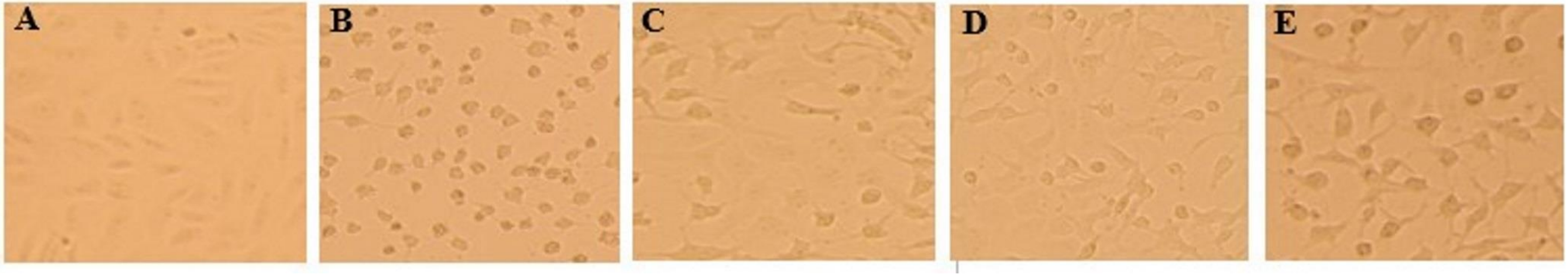

Fig S1. Protection from cytopathic effect on Vero cells using microscopy (*C. difficile* R11446 culture filtrate and zingerone were incubated at 37°C for 2 h prior to being added to Vero cell monolayers). **A**: no culture filtrate; **B:** culture filtrate only; **C:** zingerone (1.2 mg/ml); **D:** zingerone (0.6 mg/ml); **E:** zingerone (0.3 mg/ml); Microscopy × 40, Scale: 50 µm.

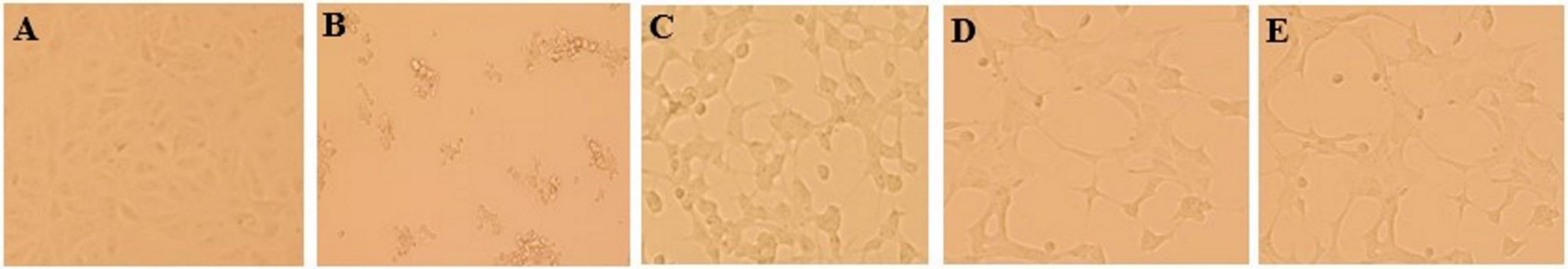

Fig S2. Protection from cytopathic effect on Vero cells using microscopy (*C. difficile* ATCC 43598 culture filtrate and zingerone were incubated at 37°C for 2 h prior to being added to Vero cell monolayers). **A**: no culture filtrate; **B:** culture filtrate only; **C:** zingerone (1.2 mg/ml); **D:** zingerone (0.6 mg/ml); **E:** zingerone (0.3 mg/ml); Microscopy × 40, Scale: 50 µm.

.

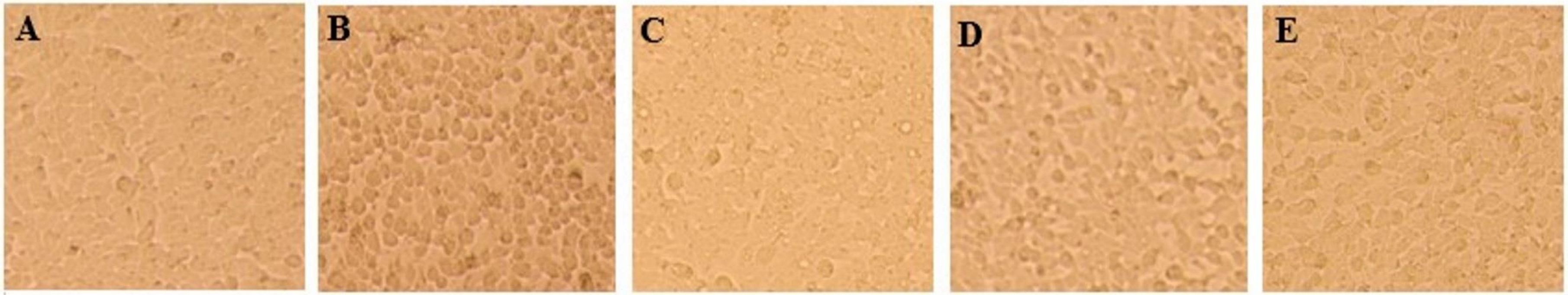

Fig S3. Protection from cytopathic effect on HT-29 cells using microscopy (*C. difficile* R11446 culture filtrate and zingerone were incubated at 37°C for 2 h prior to being added to HT-29 cell monolayers). **A**: no culture filtrate; **B:** culture filtrate only; **C:** zingerone (1.2 mg/ml); **D:** zingerone (0.6 mg/ml); **E:** zingerone (0.3 mg/ml); Microscopy × 40, Scale: 50 µm.
